# Clinical and Therapeutic Phenotypic Clustering and Prognostic Stratification in Heart Failure Patients With Atrial Fibrillation

**DOI:** 10.1002/joa3.70397

**Published:** 2026-06-18

**Authors:** Masamichi Yano, Yasuyuki Egami, Kazushi Nagai, Haya Matsubara, Ryo Muroya, Taichi Mukai, Noriyuki Kobayashi, Ayako Sugino, Masaru Abe, Hiroaki Nohara, Shodai Kawanami, Koji Yasumoto, Naotaka Okamoto, Yasuharu Matsunaga‐Lee, Masami Nishino

**Affiliations:** ^1^ Division of Cardiology Osaka Rosai Hospital Osaka Japan

## Abstract

**Background:**

Atrial fibrillation (AF) commonly coexists with heart failure (HF), but its prognostic impact remains uncertain. This study aimed to identify phenotypic subgroups of HF patients with AF and assess their clinical outcomes.

**Methods:**

A total of 407 hospitalized HF patients with AF on admission were analyzed. Key prognostic factors (age, sex, NT‐proBNP, creatinine, hemoglobin, albumin, and use of β‐blockers, renin‐angiotensin‐aldosterone system inhibitors) were standardized and subjected to hierarchical cluster analysis. The primary endpoint was a composite of all‐cause death and HF readmission.

**Results:**

Four phenotypes with distinct clinical profiles were identified. Phenotype 1 comprised younger, predominantly male patients with relatively reduced left ventricular ejection fraction but preserved systemic condition and the highest use of guideline‐directed medical therapy (GDMT), resulting in the most favorable outcomes. Phenotype 2 showed intermediate frailty and moderate GDMT use. Phenotype 3 was characterized by advanced age, systemic impairment, renal dysfunction, and anemia. Phenotype 4 represented the oldest and most frail patients with severe malnutrition and minimal GDMT use. During a median follow‐up of 612 days, Cox analysis demonstrated progressively increasing risk for the composite endpoint compared with Phenotype 1 (hazard ratios: 1.87, 3.80, and 4.60; *p* for trend < 0.001). Kaplan–Meier curves confirmed significant divergence in event‐free survival among groups.

**Conclusions:**

HF patients with AF exhibit four distinct phenotypes associated with progressively worsening outcomes. Phenotypic stratification may improve individualized risk assessment and therapeutic decision‐making in this high‐risk population.

**Trial Registration:**

Data were obtained from the Acute Heart Failure Registry in the Osaka Rosai Hospital (AURORA; UMIN000045096)

## Graphical abstract

Phenotype‐specific characteristics and potential mechanisms affecting outcomes in acute decompensated heart failure. The four phenotypes show distinct clinical profiles and associated prognoses. Phenotype 1 comprises relatively young males with reduced LVEF under optimal therapy, and Phenotype 2 includes middle‐aged females with generally favorable treatment. Phenotype 3 represents older patients with high NT‐proBNP, renal dysfunction, and anemia, whereas Phenotype 4 includes elderly, frail patients with low body mass index, malnutrition, and limited β‐blocker use. Younger phenotypes (Phenotype 1–2) benefit from effective drug therapy, likely suppressing atrial and ventricular remodeling and resulting in lower risks of heart failure–related or arrhythmic events. In contrast, older and elderly phenotypes (Phenotype 3–4) face challenges in fluid and drug management, contributing to higher risks of adverse outcomes. HF, heart failure; LVEF, left ventricular ejection fraction; NT‐proBNP, N‐terminal pro brain natriuretic peptide.

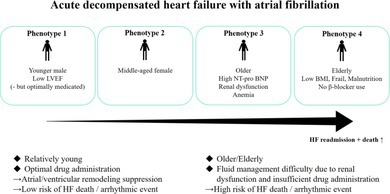

## 1 Introduction

Atrial fibrillation (AF) frequently coexists with heart failure (HF) [1], and its impact on prognosis remains a subject of ongoing debate. Several studies have reported that AF is associated with worse outcomes in patients with HF [2, 3], primarily due to the loss of atrial contraction (“atrial kick”) which can reduce cardiac output and potentially exacerbate HF symptoms [4]. However, conflicting evidence exists regarding the independent prognostic value of AF in this population. Previous studies have shown that AF does not independently predict mortality in patients with HF after adequate adjustment for confounding factors [5, 6, 7]. These findings suggest that the mere presence of AF may not uniformly confer additional risk in HF. Recent research has proposed that the prognostic significance of AF may be contingent upon underlying cardiac pathology and patient demographics. Raunso et al. reported that AF increased mortality risk only among patients with ischemic heart disease, highlighting the role of underlying etiology [8]. Similarly, Baldasseroni et al. found that AF independently predicted all‐cause mortality only in HF patients aged 75 years or younger, indicating an age‐dependent effect [9]. Despite these insights, it remains unclear which specific clinical features in HF patients with concomitant AF influence prognosis. Given the heterogeneity of this population, identifying factors that modulate risk is crucial for tailored management. In the present study, we aimed to investigate the associations between previously reported HF prognostic factors and clinical outcomes in a real‐world cohort of HF patients with AF, to better elucidate the determinants of prognosis in this subgroup.

## 2 Methods

### 2.1 Study Population

The Acute Heart Failure Registry in the Osaka Rosai Hospital (AURORA) is a single‐center study that collected the data of consecutive HF patients who were hospitalized for treatment at the Osaka Rosai Hospital (UMIN‐CTRID: UMIN000045096) [10, 11, 12]. In the present study, we included patients hospitalized for acute decompensated HF who demonstrated AF on electrocardiography at admission. The Acute Heart Failure Registry in the Osaka Rosai Hospital (AURORA) is a single‐center study that collected the data of consecutive HF patients who were hospitalized for treatment at the Osaka Rosai Hospital (UMIN‐CTRID: UMIN000045096) [10, 11, 12]. From the AURORA study, we extracted the data for HF patients who were admitted between January 2015 and December 2022. The diagnosis of HF was defined using the Framingham criteria [13]. The procedure was in accordance with the ‘Declaration of Helsinki’ and the ethical standards of the responsible committee on human experimentation. This study was approved with an exemption from requiring individual informed consent by the Osaka Rosai Hospital Ethics Committee, given its retrospective observational design.

### 2.2 Laboratory Measurement and Echocardiography

In all enrolled patients, blood samples were collected at discharge. Laboratory measurements were performed by standard methods in the clinical laboratory of our hospital. In addition, the enrolled patients underwent transthoracic echocardiography at discharge. Comprehensive echocardiographic examinations were performed by trained cardiac sonographers according to the American Society of Echocardiography guidelines [14].

### 2.3 Follow‐Up and Clinical Outcomes

Investigative cardiologists and trained research coordinators recorded the patient data including medical history, co‐morbidities, echocardiographic data, laboratory data, medications at discharge, and clinical events from the medical records. We classified the registry patients into clinically relevant phenotypic groups using unsupervised clustering based on baseline clinical characteristics and discharge therapeutic profiles related to HF prognosis and compared patient characteristics and clinical endpoints (composite of HF hospitalization and all‐cause death) between the groups.

### 2.4 Statistical Analysis

JMP 17.0.0 statistical software (SAS Institute Inc., Cary, NC) was used for the statistical analysis. A normality test was performed for continuous variables by a Shapiro–Wilk *W* test. A normal distribution was not confirmed for all variables. Categorical data were expressed as the number (percentage) and were compared using the chi‐square test. The Kruskal‐Wallis test was used for intergroup differences of continuous variables. The Bonferroni method was used to adjust *p*‐values in multiple testing. The prognostic predictability of the continuous variables for the composite endpoint was evaluated with receiver operating characteristic (ROC) curve analysis. Representative variables reflecting the multidimensional clinical status of HF patients were selected for cluster analysis. These included demographic and biological factors associated with HF prognosis, as well as discharge HF medications reflecting therapeutic implementation and treatment tolerability in real‐world clinical practice. These included patient background variables (age and sex), laboratory parameters reflecting cardiac function (N‐terminal pro brain natriuretic peptide (NT‐proBNP)) [15], renal function (creatinine) [16], nutritional status (albumin) [17], and anemia (hemoglobin) [18], as well as medication use (β‐blockers, angiotensin converting enzyme inhibitor (ACEI) / ARB, angiotensin II receptor blocker (ARB) / angiotensin receptor neprilysin inhibitor (ARNI) and mineral corticoid receptor antagonist (MRA)) [19]. Discharge HF medications were intentionally incorporated into the clustering model because prescription patterns may reflect not only therapeutic strategies but also underlying clinical conditions such as frailty, hemodynamic status, renal dysfunction, blood pressure, comorbidity burden, and treatment tolerability. To determine the optimal number of clusters, the elbow method was applied using the within‐cluster sum of squares (WSS) as an internal clustering validity index. K‐means clustering was performed by sequentially varying the number of clusters (*k* = 2 to 5). For each *k*, the total WSS across all clusters was calculated. To facilitate interpretability, the corresponding root mean square (RMS) standard deviation, derived from WSS normalized by the number of variables and observations, was also computed. The optimal number of clusters was identified at the “elbow point,” where the reduction in WSS (or RMS) began to plateau, reflecting a balance between model fit and parsimony. Subsequently, hierarchical cluster analysis was performed using the Ward's minimum variance method to classify patients based on the optimal number of clusters determined above. The results were visualized as a dendrogram to illustrate the hierarchical relationships among the clusters. To enable a direct comparison of both continuous and categorical variables across clusters, all variables were standardized prior to visualization. Continuous variables (e.g., age, log NT‐proBNP, albumin, creatinine, and hemoglobin) were standardized using *z*‐score transformation. Categorical variables (e.g., female sex, use of β‐blockers, ACEI/ARB/ARNI, and MRA) were expressed as proportions within each cluster and subsequently standardized using the same *z*‐score transformation. These standardized values were then used to generate radar charts, allowing intuitive visualization of the characteristic clinical profiles of each cluster. Kaplan–Meier curves were used for the comparisons and statistical significance was determined using the Log‐rank test. Cumulative incidence functions were estimated for the composite endpoint of HF readmission and all‐cause death, accounting for competing risks. Cumulative incidence of events was compared between groups using Gray's test, accounting for competing risks. Multivariable Cox proportional hazards analysis was performed to compare the hazard ratio of the composite endpoint and adjusted hazard ratios (HR) and 95% confidence intervals (CI) were calculated.

## 3 Results

### 3.1 Patients Characteristics

Between January 2015 and December 2022, a total of 2325 patients were enrolled from the AURORA registry. This study included 407 patients after excluding 1065 patients with incomplete discharge data and 853 individuals who did not exhibit AF during hospitalization. The baseline characteristics of the enrolled AF patients are shown in Table 1.

**TABLE 1:** Baseline characteristics.

	Overall population (*n* = 407)	Phenogroups			
		Phenotype 1 (*n* = 101)	Phenotype 2 (*n* = 151)	Phenotype 3 (*n* = 106)	Phenotype 4 (*n* = 49)
*Clinical data*					
Age, years	80 [75, 85]	74 [62, 80]	80 [75, 84]^a^	83 [78, 87]^a^, ^c^	87 [84, 90]^a^, ^c^, ^e^
Female	197 (48.4)	3 (3.0)	124 (82.1)^a^	36 (34.0)^a^, ^c^	34 (48.4)^a^, ^e^
Body mass index, kg/m^2^	23.7 [21.1, 27.0]	24.6 [22.4, 28.1]	24.4 [21.1, 27.4]	23.2 [21.3, 25.6]^a^, ^d^	20.9 [19.1, 23.8]^a^, ^c^, ^e^
Clinical frail scale	3 [2, 5]	3 [2, 3]	3 [2, 5]^a^	4 [3, 5]^a^, ^c^	5 [4, 7]^a^, ^c^, ^e^
Smoking (current)	53 (13.0)	30 (29.7)	11 (7.3)^a^	11 (10.4)^a^	1 (2.0)^a^
Hypertension	290 (71.3)	66 (65.4)	108 (71.5)	85 (80.2)	31 (63.3)
Diabetes	111 (27.3)	26 (25.7)	40 (26.5)	32 (30.2)	13 (26.5)
Dyslipidemia	127 (31.2)	32 (31.7)	56 (37.1)	31 (29.3)	8 (16.3)
History of PCI	59 (14.5)	14 (13.9)	18 (11.9)	23 (21.7)	4 (8.2)
History of CABG	27 (6.6)	7 (6.9)	1 (0.7)^b^	17 (16.0)^c^	2 (4.1)
History of valvular surgery	41 (10.1)	5 (5.0)	20 (13.3)	11 (10.4)	5 (10.2)
Catheter ablation for atrial fibrillation after admission	77 (18.9)	33 (32.7)	32 (21.2)^a^	10 (9.4)^a^, ^c^	2 (4.1)^a^, ^c^, ^e^
*Laboratory data (at discharge)*					
Albumin, g/dl	3.5 [3.2, 3.8]	3.8 [3.6, 4.0]	3.6 [3.3, 3.8]^a^	3.2 [3.0, 3.7]^a^, ^c^	3.1 [2.9, 3.2]^a^, ^c^, ^e^
Blood urea nitrogen, mg/dl	26 [20, 37]	22 [18, 29]	24 [18, 32]	36 [24, 50]^a^, ^c^	28 [25, 49]^a^, ^c^
Creatinine, mg/dl	1.09 [0.86, 1.53]	1.06 [0.87, 1.28]	0.92 [0.73, 1.17]^a^	1.66 [1.28, 2.30]^a^, ^c^	1.02 [0.90, 1.50]^c^, ^e^
Hemoglobin, g/dl	11.9 [10.5, 13.6]	14.6 [13.3, 16.2]	12.0 [11.0, 13.5]^a^	10.5 [9.4, 11.3]^a^, ^c^	10.6 [9.2, 11.3]^c^
Log NT‐proBNP, pg/ml	3.21 [2.87, 3.60]	2.98 [2.72, 3.29]	3.08 [2.76, 3.30]	3.62 [3.42, 3.84]^a^, ^c^	3.36 [3.15, 3.75]^a^, ^c^, ^e^
*Echocardiographic parameters*					
LVDd, mm	51 [46, 56]	54 [48, 60]	49 [44, 55]^a^	51 [46, 56]^a^	48 [41, 55]^a^
LVDs, mm	35 [29, 45]	43 [33, 49]	33 [29, 40]^a^	34 [29, 43]^a^	30 [26, 41]^a^, ^e^
LVEF, %	54 [38, 65]	41 [31, 58]	58 [45, 65]^a^	55 [40, 64]^a^	64 [48, 71]^a^, ^d^, ^e^
LA diameter, mm	50 [46, 55]	50 [47, 54]	50 [46, 55]	51 [47, 57]^b^	48 [44, 52]^e^
Septal E/e'	15.8 [12.2, 20.2]	13.6 [10.5, 17.9]	16.7 [12.9, 21.2]^a^	17.2 [12.6, 21.3]^a^	16.3 [13.4, 20.2]^a^
Inferior vena cava diameter, mm	16 [13, 20]	15 [11, 18]	15 [13, 19]^a^	17 [15, 22]^c^	16 [12, 20]
Severe MR	85 (20.9)	13 (12.9)	35 (23.2)	30 (28.3)	7 (14.3)
Severe TR	102 (27.3)	10 (10.5)	40 (29.0)^a^	31 (32.6)^a^	21 (45.7)^a^
*Medications*					
ACEI/ARB/ARNI	218 (53.6)	69 (68.3)	81 (53.6)	49 (46.2)^b^	19 (38.8)^a^
β‐blocker	298 (73.2)	79 (78.2)	130 (86.1)	89 (84.0)	0 (0)^a^, ^c^, ^e^
Mineral corticoid receptor antagonist	195 (47.9)	61 (60.4)	64 (42.4)^b^	55 (51.9)	15 (30.6)^a^
Loop diuretics	356 (87.5)	84 (83.2)	139 (92.1)	94 (88.7)	39 (79.6)
Statin	149 (36.6)	33 (32.7)	65 (43.1)	42 (39.6)	9 (18.4)^d^
Anticoagulants	335 (82.3)	95 (94.1)	126 (83.4)	83 (78.3)^a^	31 (63.3)^a^, ^d^
Antiplatelet drugs	113 (27.8)	34 (33.7)	33 (21.9)	37 (18.4)	9 (18.4)

*Note:* Categorical variables are presented as numbers (percentage). Continuous data are presented as the median (interquartile range). Categorical variables are presented as numbers (percentage).

Abbreviations: ACEI, angiotensin converting enzyme inhibitor; ARB, angiotensin II receptor blocker; ARNI, angiotensin receptor neprilysin inhibitor; CABG, coronary artery bypass grafting; LA, left atrium; LVDd, left ventricular end‐diastolic diameter left ventricular diameter; LVDs, left ventricular end‐systolic diameter; LVEF, left ventricular ejection fraction; MR, mitral regurgitation; NT‐proBNP, N‐terminal pro brain natriuretic peptide; PCI, percutaneous coronary intervention; TR, tricuspid regurgitation.

^a^ *p* < 0.01 versus Phenotype 1.

^b^ *p* < 0.05 versus Phenotype 1.

^c^ *p* < 0.01 versus Phenotype 2.

^d^ *p* < 0.05 versus Phenotype 2.

^e^ *p* < 0.01 versus Phenotype 3.

### 3.2 Phenotypic Clustering of Atrial Fibrillation Patients With Heart Failure

**FIGURE 1:**
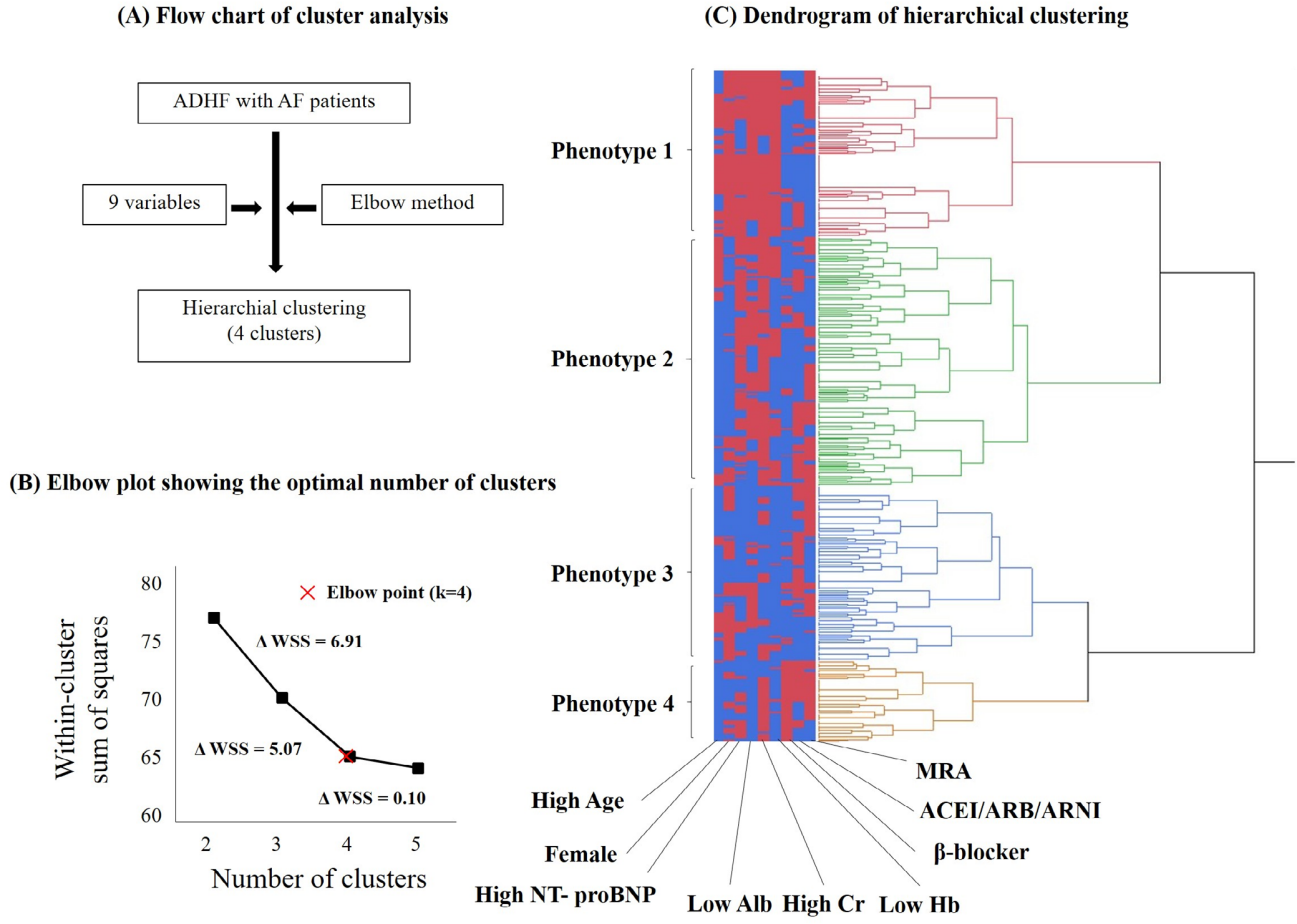
Cluster analysis of the study population. (A) Flow chart summarizing the clustering analysis process: Nine factors were collected from the study population, WSS was calculated to determine the optimal number of clusters, and hierarchical clustering was performed to classify the population into four clusters. (B) Elbow plot showing the WSS used to determine the optimal number of clusters. Nine factors were included in the analysis, and the point of inflection indicates the appropriate number of clusters. (C) Dendrogram of hierarchical clustering, showing the division of the population into four distinct clusters. ACEI, angiotensin converting enzyme inhibitor; ADHF, acute decompensated heart failure; AF, atrial fibrillation; Alb, albumin; ARB, angiotensin II receptor blocker; ARNI, angiotensin receptor neprilysin inhibitor; Cr, creatinine; Hb, hemoglobin; MRA, Mineral corticoid receptor antagonist; NT‐proBNP, N‐terminal pro brain natriuretic peptide; WSS, within‐cluster sum of squares.

Optimal cut‐off values for key continuous variables were determined by ROC analysis: age 77 years, log NT‐proBNP 3.32 pg/mL, creatinine 1.52 mg/dL, and hemoglobin 12.4 g/dL (Figure S1). To identify the optimal number of clusters, the elbow method was applied using the WSS as an internal validity index. K‐means clustering was sequentially performed for *k* = 2 to 5, yielding WSS values of 77.08, 70.17, 65.10, and 64.10, respectively. The optimal number of clusters was determined to be four, as the reduction in WSS began to plateau at *k* = 4, indicating a balance between model fit and parsimony (Figure 1). Based on this optimal cluster number, hierarchical clustering was performed using nine variables—age ≥ 77 years, female sex, log NT‐proBNP ≥ 3.32 pg/mL, creatinine ≥ 1.52 mg/dL, hemoglobin < 12.4 g/dL, and the use of β‐blockers, ACEI/ARB/ARNI, and MRAs—to define four distinct phenotypes (Figure 1).

### 3.3 Characteristics of Four Phenotypes

**FIGURE 2:**
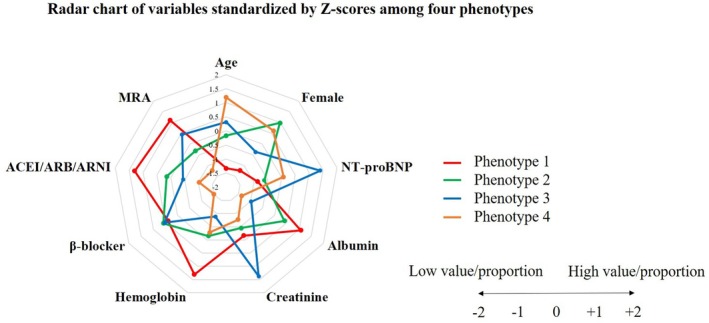
Radar charts showing characteristic clinical profiles of the four phenotypes. Nine factors, including continuous variables (age, log NT‐proBNP, albumin, creatinine, and hemoglobin) and categorical variables (female sex, use of β‐blockers, ACEI/ARB/ARNI, and MRA), were used to generate the radar charts. Continuous variables were standardized using *z*‐score transformation. Categorical variables were expressed as proportions within each phenotype and then standardized using the same *z*‐score transformation. These standardized values were plotted to enable direct comparison across Phenotypes 1–4, providing an intuitive visualization of the differences in clinical profiles among the clusters. ACEI, angiotensin converting enzyme inhibitor; ARB, angiotensin II receptor blocker; ARNI, angiotensin receptor neprilysin inhibitor; MRA, Mineral corticoid receptor antagonist; NT‐proBNP, N‐terminal pro brain natriuretic peptide.

The baseline characteristics of the four phenotypes are shown in Table 1. Patients in Phenotype 1 were the youngest (median age 74 years) and predominantly male (97%), with the lowest frailty scores and the highest serum albumin and hemoglobin levels. Renal function was relatively preserved, and the prescription rates of evidence‐based therapies, including β‐blockers (78%), ACEI/ARB/ARNI (68%), and MRAs (60%), were the highest among all groups. Phenotype two patients were older (median 80 years), predominantly female (82%), and exhibited mildly increased frailty and moderate anemia despite preserved renal function and nutritional status. The use of guideline‐directed medical therapy (GDMT) was moderate, with β‐blockers prescribed in 86% and ACEI/ARB/ARNI in 54% of patients. Phenotype 3 was characterized by advanced age (median 83 years), pronounced frailty, and evidence of systemic deterioration, including hypoalbuminemia, renal dysfunction (median creatinine 1.66 mg/dL), and anemia. Although the prescription of β‐blockers (84%) and MRAs (52%) remained relatively frequent, the use of ACEI/ARB/ARNI was lower (46%). In contrast, Phenotype 4 comprised the oldest patients (median 87 years) with the highest frailty burden and the lowest albumin and hemoglobin levels. Despite having relatively preserved renal function, this group exhibited extremely low rates of GDMT use—particularly a complete absence of β‐blocker prescription (0%) and reduced use of ACEI/ARB/ARNI (39%) and MRAs (31%). Radar chart analysis based on standardized (Z‐score–transformed) variables revealed distinct clinical profiles across the four phenotypes (Figure 2). Phenotype 1 was characterized by younger age (−1.33), male predominance (−1.23), preserved nutritional and hematologic status (albumin 1.07, hemoglobin 1.31), and frequent use of evidence‐based therapies (β‐blockers 0.39, ACEI/ARB/ARNI 1.31, MRA 1.10). Phenotype 2 showed intermediate characteristics with modest frailty and moderate treatment intensity. Phenotype 3 demonstrated systemic impairment with elevated NT‐proBNP (1.40), higher creatinine (1.38), and lower albumin (−0.97). In contrast, Phenotype 4 represented the oldest and most frail group, with marked hypoalbuminemia (−1.37), low hemoglobin (−0.28), and minimal GDMT use (β‐blockers −1.50, ACEI/ARB/ARNI −1.03, MRA −1.23).

### 3.4 Incidence of the Composite Endpoint Among the Four Phenotypes

**FIGURE 3:**
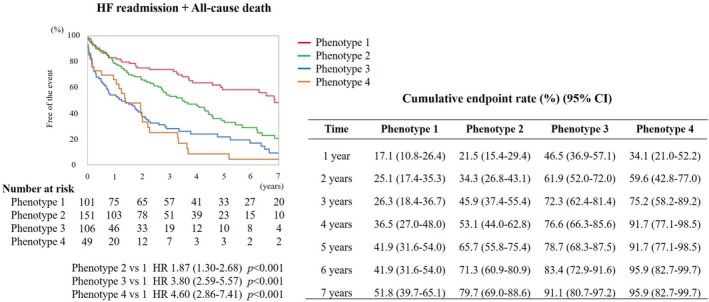
Clinical outcomes according to the four phenotypes. Kaplan–Meier curves for the composite endpoint of heart failure rehospitalization and all‐cause mortality are shown for Phenotypes 1–4. Cumulative event rates for each phenotype are indicated on the curves. Cox proportional hazards analysis was performed to estimate HR and 95% CI for the risk of the composite endpoint across phenotypes, with one phenotype designated as the reference group. This figure illustrates differences in event‐free survival and relative risk among the four phenotypes. CI, confidence interval; HF, heart failure; HR, hazard ratio.

The median follow‐up duration was 612 days. The composite endpoint of HF hospitalization and all‐cause mortality occurred in 47 of 102 patients (47.1%) in Phenotype 1, 86 of 151 (57.0%) in Phenotype 2, 74 of 106 (69.8%) in Phenotype 3, and 30 of 49 (61.2%) in Phenotype 4. Among these, all‐cause mortality was observed in 22 of 102 patients (21.6%) in Phenotype 1, 50 of 151 (33.1%) in Phenotype 2, 48 of 106 (45.2%) in Phenotype 3, and 16 of 49 (32.7%) in Phenotype 4. The number of deaths with unknown causes was 10/22 in Phenotype 1, 30/50 in Phenotype 2, 18/48 in Phenotype 3, and 5/16 in Phenotype 4. Deaths that could be clearly classified as HF–related or arrhythmia‐related were as follows: HF–related deaths, 4/22 in Phenotype 1, 8/50 in Phenotype 2, 12/48 in Phenotype 3, and 5/16 in Phenotype 4; arrhythmia‐related deaths, 0/22 in Phenotype 1, 0/50 in Phenotype 2, 3/48 in Phenotype 3, and 3/16 in Phenotype 4. Kaplan–Meier analysis demonstrated that Phenotype 1 had the lowest risk of the composite endpoint of HF hospitalization and all‐cause mortality, with progressively worse outcomes observed in Phenotypes 2, 3, and 4 (Phenotype 2 vs. 1: HR 1.87 [95% CI 1.30–2.68], *p* < 0.001; Phenotype 3 vs. 1: HR 3.80 [95% CI 2.59–5.57], *p* < 0.001; Phenotype 4 vs. 1: HR 4.60 [95% CI 2.86–7.41], *p* < 0.001) (Figure 3). In the multivariable Cox proportional hazards analysis, phenotype was independently associated with the composite outcome. Compared with phenotype 1, phenotype 2 (HR 1.91, 95% CI 1.21–3.04; *p* = 0.006), phenotype 3 (HR 3.22, 95% CI 1.96–5.26; *p* < 0.001), and phenotype 4 (HR 3.63, 95% CI 1.91–6.87; *p* < 0.001) were all associated with a significantly higher risk. Catheter ablation was associated with a lower risk of the composite endpoint (HR 0.36, 95% CI 0.21–0.62; *p* < 0.001), whereas hypertension, diabetes mellitus, anticoagulant use, left ventricular ejection fraction, left atrial diameter, and septal E/e' were not significantly associated with outcomes (Table 2).

**TABLE 2:** Cox proportional hazard analysis for the composite of HF readmission and all‐cause death.

Variables	HR	95% CI	*p*
Phenotypes			
Phenotype 1 (Reference)	1.00	—	—
Phenotype 2	1.91	1.21–3.04	0.006
Phenotype 3	3.22	1.96–5.26	< 0.001
Phenotype 4	3.63	1.91–6.87	< 0.001
Hypertension	1.25	0.84–1.86	0.281
Diabetes mellitus	1.23	0.86–1.75	0.257
Catheter ablation	0.36	0.21–0.62	< 0.001
Anticoagulant	0.87	0.56–1.35	0.527
Left ventricular ejection fraction	1.00	0.9–1.012	0.911
Left atrial diameter	1.02	0.99–1.04	0.067
Septal E/e'	1.00	0.97–1.02	0.712

Abbreviations: CI, confidence interval; HF, heart failure; HR, hazard ratio.

## 4 Discussion

### 4.1 Main Findings

In the present study, we classified AF patients with HF into four distinct phenotypes based on clinical and laboratory variables using cluster analysis. These phenotypes differed in age, frailty, systemic comorbidities, and the use of GDMT, enabling effective risk stratification for the composite endpoint of HF hospitalization and all‐cause mortality. Notably, younger, less frail patients receiving optimal therapy had the most favorable outcomes, whereas older, frail patients with systemic impairment and limited therapy experienced worse prognosis. These findings highlight the heterogeneity of AF patients with HF and underscore the importance of comprehensive patient assessment and personalized management strategies.

### 4.2 Prognostic Impact of Atrial Fibrillation in Heart Failure Patients

A recent meta‐analysis of published randomized clinical trials and observational studies confirmed reports that AF is associated with worse outcomes in HF patients. In this analysis which included over 32 000 patients, AF in HF patients was associated with a significantly increased risk of death [20]. The loss of atrial contraction, irregular rhythm, thrombus formation, and increased sympathetic nervous system activity are all potential mechanisms through which AF negatively impacts the prognosis of HF [21]. These factors contribute to the exacerbation of HF symptoms, deterioration of cardiac function, and increased risk of adverse outcomes, including mortality and hospitalization. Specifically, the absence of atrial contraction compromises ventricular filling, while the irregular rhythm leads to hemodynamic instability [21]. Additionally, AF is associated with a higher incidence of thromboembolic events, further complicating the clinical management of HF patients. The heightened sympathetic activity associated with AF may exacerbate the underlying pathophysiology of HF, leading to a vicious cycle of deterioration in cardiac function. However, it is still unclear whether it is the arrhythmia itself or the associated comorbidities and risk factors that convey the extra risk. Some investigations have shown that AF does not independently predict mortality in HF patients when confounding factors are adequately adjusted [5, 6, 7]. These results may indicate that the adverse prognostic impact of AF is largely reduced when patients receive adequate rate control, anticoagulation, and optimized HF therapy, suggesting that AF itself is not a major determinant of outcome in well‐managed HF populations. These findings suggest that AF has a negative impact on the prognosis of HF patients; however, the extent of this impact may vary depending on the patient's treatment status and coexisting risk factors. Therefore, risk stratification is considered crucial in patients with AF and HF. In this context, we focused on the need for risk stratification in patients with AF and HF, and employed cluster analysis to investigate the characteristics of different risk groups.

### 4.3 Distinct Phenotypes and Clinical Outcomes

In this study, we performed a clustering analysis of patients with acute HF and identified four distinct phenotypes with differing clinical characteristics and prognoses. Notably, despite exhibiting the lowest left ventricular ejection fraction (LVEF), patients in Phenotype 1 demonstrated a significantly lower incidence of the composite endpoint compared with other groups. This subgroup was characterized by younger age, lower clinical frail scores, preserved nutritional and renal status, and the highest prescription rates of evidence‐based therapies, including β‐blockers, renin–angiotensin–aldosterone system inhibitors, and MRAs. These findings suggest that patients with HF with reduced ejection fraction (HFrEF) who have fewer systemic comorbidities and receive optimal medical therapy may achieve favorable outcomes, thereby reinforcing the prognostic importance and efficacy of GDMT for HFrEF. Even in the presence of AF, the adverse effects of AF on outcomes may be reduced by appropriate evidence‐based treatment for HFrEF. In contrast, other phenotypes exhibited preserved LVEF but less favorable outcomes, highlighting the heterogeneity of HF presentations. In Phenotype 2, although LVEF was preserved (mean LVEF = 58%), the incidence of MRA use was low, and mild frailty and anemia were frequently observed. These findings indicate that suboptimal therapeutic intervention may have contributed to the moderately unfavorable prognosis in this group. Although NT‐proBNP levels were relatively low, the preserved LVEF did not necessarily translate to a better outcome, suggesting that early management of frailty and extracardiac comorbidities is crucial in HF with preserved ejection fraction (HFpEF). Phenotype 3 represented a more complex HFpEF phenotype, characterized by older age, higher NT‐proBNP levels, malnutrition, anemia, renal dysfunction, and severe frailty, all of which reflect systemic vulnerability. Although LVEF was preserved (mean LVEF = 55%), deterioration in overall systemic condition rather than cardiac function alone appeared to drive adverse outcomes. These findings emphasize the importance of a comprehensive assessment that includes non‐cardiac factors such as renal function, hematologic indices, and nutritional status. In contrast, Phenotype 4 showed the highest LVEF (mean LVEF = 64%) but the poorest prognosis. This group was characterized by advanced age, frailty, malnutrition, renal dysfunction, and remarkably low prescription rates of β‐blockers, renin–angiotensin–aldosterone system inhibitors, and MRAs. The combination of therapeutic limitations and systemic frailty likely contributed to the markedly poor outcomes observed in this phenotype. HFpEF is known to be associated with multiple systemic factors such as advanced age, female sex, obesity, diabetes, and renal dysfunction [22], and is frequently accompanied by AF due to structural and electrical remodeling of the atria. The presence of AF leads to the loss of atrial contraction and irregular ventricular rhythm, which can directly increase filling pressures and exacerbate congestion in HFpEF patients who already have impaired diastolic function. Indeed, previous reports have shown that among elderly patients with AF, those with concomitant HF represent a frail subgroup with impaired physical and cognitive function and a higher risk of progression to frailty within a short period [23]. Furthermore, β‐blocker use has been associated with reduced mortality in patients with AF and HF, whereas such benefits were not observed in AF patients without HF [24]. These findings reinforce the concept that the combination of “advanced age, frailty, malnutrition, renal dysfunction, and under‐prescription of evidence‐based therapies” observed in Phenotype 4 constitutes a multilayered risk structure leading to adverse outcomes. Previous studies have similarly reported that AF does not significantly affect prognosis in HFrEF, whereas it is strongly associated with poor outcomes in HFpEF [25], which is consistent with our findings. Thus, in HFpEF, AF may not merely represent a rhythm disturbance but may instead reflect an interplay of systemic vulnerability and therapeutic limitations, exerting a substantial impact on prognosis. Collectively, these observations underscore that heterogeneity in patient profiles, rather than LVEF alone, critically determines outcomes in acute HF. These results highlight the need for comprehensive management strategies and optimized medical therapy in this high‐risk population. An important consideration of this study is the incremental value of phenotypic clustering compared with established HF risk scores such as MAGGIC and GWTG‐HF [26, 27]. While these validated scores provide robust prognostic information in general HF populations, they were not specifically designed for patients with AF, and their applicability in AF‐dominant HF cohorts remains uncertain. In addition, conventional risk scores do not account for AF‐specific characteristics, including arrhythmia burden, rhythm control strategies, or AF‐related atrial remodeling, which may contribute to clinical heterogeneity in this population. Therefore, the present phenotypic approach should be interpreted as a complementary framework rather than a replacement for established risk models. Clinically, the phenotypes identified in this study may not directly alter guideline‐directed therapy; however, they may provide additional granularity for risk stratification, helping clinicians to identify patients with particularly high‐risk profiles within AF‐associated HF.

### 4.4 Clinical Implication

Our findings highlight that AF does not uniformly worsen the prognosis of HF patients but interacts with systemic vulnerability and therapeutic adequacy. Cluster‐based phenotyping enables more precise risk stratification, identifying subgroups who may benefit from aggressive optimization of GDMT and those requiring multidisciplinary care addressing frailty, malnutrition, and renal dysfunction. Particularly in HFpEF, AF may represent a marker of systemic decline rather than a purely electrical disorder, emphasizing the need for comprehensive management strategies beyond conventional HF therapy.

### 4.5 Study Limitation

This study has several limitations. First, it was a retrospective, single‐center study conducted at a tertiary care hospital, which may limit the generalizability of our findings. Second, many patients were excluded because of incomplete discharge data, potentially introducing selection bias. Baseline characteristics, including age and sex, were similar between enrolled and excluded patients (Table S1). However, excluded patients had more diabetes, prior percutaneous coronary intervention, renal dysfunction, and anemia, indicating a higher burden of ischemic and metabolic comorbidities. In contrast, enrolled patients more frequently showed AF‐related HF features, including larger left atrial diameter and greater use of β‐blockers and anticoagulants, suggesting that AF contributed more substantially to HF pathophysiology in this cohort. Third, although nine key factors were used for cluster analysis, the potential influence of other unmeasured variables cannot be entirely excluded. Fourth, AF was defined based on its presence on electrocardiography at the time of hospital admission for heart failure, without consideration of AF duration or temporal pattern. Although this pragmatic definition reflects real‐world clinical practice, it does not allow differentiation between AF subtypes, such as paroxysmal, persistent, or long‐standing persistent AF. Because AF temporal patterns were not prospectively collected in the registry, AF subtype classification and related subgroup analyses could not be performed. Given the potential prognostic relevance of AF classification in patients with heart failure, this represents an important limitation of the present study. Fifth, catheter ablation was not evenly distributed across phenotypic groups and was strongly associated with baseline clinical characteristics. Therefore, potential interdependence between treatment exposure and phenotypic classification should be considered when interpreting their respective associations with outcomes. Finally, in Japan, sodium–glucose cotransporter 2 (SGLT2) inhibitors were approved for HFrEF only from December 2020. Therefore, the prescription and prognostic impact of SGLT2 inhibitors could not be evaluated in this study. Finally, external validation is required to confirm the reproducibility of our findings. Despite these limitations, standardized follow‐up and comprehensive evaluation of four distinct phenotypes enhance the reliability and clinical relevance of our results.

## 5 Conclusion

HF patients with AF exhibit substantial heterogeneity, which can be categorized into four distinct phenotypes with differing clinical characteristics, comorbidities, and utilization of GDMT. Phenotypic stratification effectively discriminates risk for the composite endpoint of HF hospitalization and all‐cause mortality, with younger, less frail patients receiving optimal therapy demonstrating the most favorable outcomes, and older, frail patients with systemic impairment and limited therapy showing the highest risk. These findings underscore the utility of phenotype‐based assessment in guiding individualized management and optimizing prognostic evaluation in HF patients with AF.

## Funding

The authors have nothing to report.

## Disclosure

The authors have nothing to report.

## Conflicts of Interest

The authors declare no conflicts of interest.

## Supporting information


**Table S1:** Baseline characteristics.

**Figure S1:** ROC curve analysis for predicting the composite outcome of HF hospitalization and all‐cause death by (A) age, (B) log NT‐proBNP, (C) albumin, (D) creatinine, and (E) hemoglobin. AUC, area under the curve; HF, heart failure; ROC, receiver operating curve.

## Data Availability

The data sets analyzed in this study will be shared upon reasonable request to the corresponding author after the approval of the Institutional Review Board in Osaka Rosai Hospital.
