# Supplementary material for: Clinical and Therapeutic Phenotypic Clustering and Prognostic Stratification in Heart Failure Patients With Atrial Fibrillation
Source: J Arrhythm. 2026 Jun 18;42(3):e70397. doi: 10.1002/joa3.70397 (PMC13279884; doi:10.1002/joa3.70397)
Supplement: Supplementary file 1 — Table S1: Baseline characteristics. [file JOA3-42-e70397-s001.docx]

**Supplementary Table 1** Baseline characteristics

|  | Enrolled patients (n=407) | Excluded patients (n=1065) | P value |  |
| --- | --- | --- | --- | --- |
|  |  |  |  |  |
| **Clinical data** |  |  |  |  |
| Age, yrs | 80 [75, 85] | 81 [74, 87] | 0.142 |  |
| Female | 197 (48.4) | 506 (47.5) | 0.760 |  |
| Body mass index, kg/m^2^ | 23.6 [21.1, 27.0] | 22.8 [20.4, 25.6] | <0.001 |  |
| Clinical frail scale | 3 [2, 5] | 3 [2. 6] | 0.052 |  |
| Smoking (current) | 53 (13.0) | 116 (10.9) | 0.257 |  |
| Hypertension | 290 (71.3) | 741 (69.6) | 0.546 |  |
| Diabetes | 111 (27.3) | 394 (37.0) | <0.001 |  |
| Dyslipidemia | 127 (31.2) | 363 (34.1) | 0.289 |  |
| History of PCI | 59 (14.5) | 206 (19.4) | 0.030 |  |
| History of CABG | 27 (6.6) | 80 (7.5) | 0.559 |  |
| History of valvular surgery | 41 (10.1) | 125 (11.8) | 0.364 |  |
| **Laboratory data (at discharge)** |  |  |  |  |
| Albumin, g/dl | 3.5 [3.2, 3.8] | 3.3 [2.9, 3.7] | <0.001 |  |
| Blood urea nitrogen, mg/dl | 26 [20, 37] | 29 [21, 46] | <0.001 |  |
| Creatinine, mg/dl | 1.09 [0.86, 1.53] | 1.24 [0.90, 1.93] | <0.001 |  |
| Hemoglobin, g/dl | 11.9 [10.5, 13.6] | 10.9 [9.5, 12.6] | <0.001 |  |
| Log NT-proBNP, pg/ml | 3.21 [2.87, 3.60] | 3.27 [2.94, 3.70] | 0.072 |  |
| **Echocardiographic parameters** |  |  |  |  |
| LVDd, mm | 51 [46, 56] | 52 [47, 58] | 0.104 |  |
| LVDs, mm | 35 [29, 45] | 37 [30, 46] | 0.120 |  |
| LVEF, % | 54 [38, 65] | 50 [37, 64] | 0.199 |  |
| LA diameter, mm | 50 [46, 55] | 48 [44, 53] | <0.001 |  |
| Septal E/e’ | 15.8 [12.2, 20.2] | 16.9 [13.1, 22.3] | 0.002 |  |
| Inferior vena cava diameter, mm | 16 [13, 20] | 16 [13, 20] | 0.913 |  |
| Severe MR | 85 (20.9) | 210 (22.9) | 0.886 |  |
| Severe TR | 102 (27.3) | 222 (24.2) | 0.167 |  |
| **Medications** |  |  |  |  |
| ACEI/ARB/ARNI | 218 (53.6) | 539 (50.7) | 0.319 |  |
| β-blocker | 298 (73.2) | 639 (60.1) | <0.001 |  |
| Mineral corticoid receptor antagonist | 195 (47.9) | 423 (39.8) | 0.005 |  |
| Loop diuretics | 356 (87.5) | 840 (79.0) | <0.001 |  |
| Statin | 149 (36.6) | 397 (37.3) | 0.803 |  |
| Anticoagulants | 149 (36.6) | 382 (35.9) | 0.801 |  |
| Antiplatelet drugs | 335 (82.3) | 27 (6.6) | <0.001 |  |

Categorical variables are presented as numbers (percentage). Continuous data are presented as the median (interquartile range). Categorical variables are presented as numbers (percentage). ACEI, angiotensin converting enzyme inhibitor; ARB, angiotensin II receptor blocker; ARNI, angiotensin receptor neprilysin inhibitor: NT-proBNP, N-terminal pro brain natriuretic peptide; CABG, coronary artery bypass grafting; LA, left atrium; LVDd, left ventricular end-diastolic diameter left ventricular diameter; LVDs, left ventricular end-systolic diameter; LVEF, left ventricular ejection fraction; PCI, percutaneous coronary intervention; MR, mitral regurgitation; TR, tricuspid regurgitation.

All variables including clinical data, laboratory data, echocardiographic parameters, and medications were available in enrolled patients. Clinical data were available for all patients in excluded patients. Laboratory data for albumin, blood urea nitrogen, creatinine, and hemoglobin were available in 1026 excluded patients. NT-proBNP data were available in 33 excluded patients. Echocardiographic parameters at discharge were available in 919 excluded patients. Discharge medication data were available for all patients in excluded patients.
