# Supplementary figures and images for: Clinical and Therapeutic Phenotypic Clustering and Prognostic Stratification in Heart Failure Patients With Atrial Fibrillation
Source: J Arrhythm. 2026 Jun 18;42(3):e70397. doi: 10.1002/joa3.70397 (PMC13279884; doi:10.1002/joa3.70397)

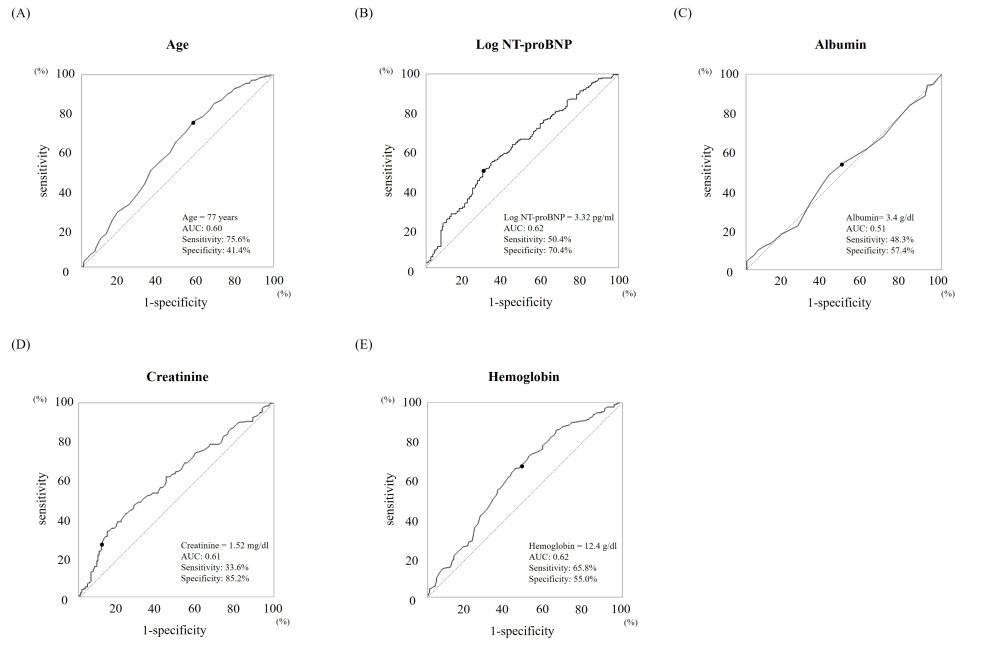

Supplement: Supplementary file 2 — Figure S1: ROC curve analysis for predicting the composite outcome of HF hospitalization and all‐cause death by (A) age, (B) log NT‐proBNP, (C) albumin, (D) creatinine, and (E) hemoglobin. AUC, area under the curve; HF, heart failure; ROC, receiver operating curve. [file JOA3-42-e70397-s002.tif]
